# Editorial: Insights in Protein Chemistry and Enzymology: 2021

**DOI:** 10.3389/fmolb.2022.944975

**Published:** 2022-06-20

**Authors:** Hang Fai Kwok, Qi Zhang

**Affiliations:** ^1^ Cancer Centre, Faculty of Health Sciences, University of Macau, Macau, Macau SAR, China; ^2^ Department of Biomedical Sciences, Faculty of Health Sciences, University of Macau, Macau, Macau SAR, China; ^3^ Department of Chemistry, Fudan University, Shanghai, China

**Keywords:** protein chemistry design and folding, enzymology characterization, structural biology, drug discovery, proteomic analyses, inhibitor

As we are entering the third decade of the 21st Century, especially in the last few years, the achievements made by biochemists, chemists and molecular biologists have been exceptional, leading to significant advancements in the fast-growing field of Protein Chemistry and Enzymology. In this Frontiers Research Topic, we have edited five manuscripts (three original research articles and two review articles) that cover a broad range of research in protein chemistry and enzymology, showcasing the latest advancements at the forefront of molecular bio-sciences and the importance of this field for the basic and clinical research.

An important aspect of this Research Topic is to identify novel protein-protein/protein-ligand interactions using state-of-the-art structural biology tools such as X-ray crystallography and NMR spectroscopy. Pan et al. solved the crystal structures of BCMO-1 and BCMO-2 that belong to the carotenoid cleavage dioxygenases (CCD) family, a family of non-heme iron-containing dioxygenase enzymes catalyzing the oxidative cleavage of carotenoid substrates. Roberts and Hedstrom review the use of high-resolution field cycling NMR to interrogate the dynamics of protein-bound ligands. This powerful technique can reveal new binding models and interactions, which are usually not considered part of the reaction coordinate but actually could be active participants in catalysis.

Protein phosphorylation has become a central focus of drug discovery as the result of the identification and validation of promising therapeutic targets such as protein kinases, protein phosphatases, and phosphoprotein binding domains. Wang et al. carried out an extensive proteomic analysis for the pathological site of human Rotator cuff tendinopathy (RCT) patients, showing that the majority of proteins with upregulated phosphorylation sites (p-sites) are related to neutrophil-mediated immunity whereas the down-regulated p-sites are mainly involved in muscle development. The authors also built a weighted kinase-site phosphorylation network to identify potentially core kinases, from which serine/threonine-protein kinase 39 (STLK3) and mammalian STE20-like protein kinase 1 (MST1) were proposed to be positively correlated with the activation of the Wnt pathway.

Contact-dependent growth inhibition (CDI) is an important mechanism for bacterial competition and communication. Cuthbert et al. review the most recent studies in the characterization of CDI, focusing on the diverse array of CDI toxin/immunity complex structures and the distinct toxin functions. The authors also discuss how these diverse toxin/immunity complexes could be harnessed to fight human diseases by utilizing bacterial CDI inhibition effectors.

Finally, Jia et al. performed an interesting proteomic study to comparatively analyze the salivary samples from different glucose control levels. This analysis identified differentially expressed proteins associated with regulated pathways and evaluated potential biomarkers in relation to type 2 diabetes mellitus (T2DM). Three proteins TXNDC17, ZG16B, and FAM3D were found to be potential biomarkers to distinguish high and low blood glucose states and further investigate hyperglycemia and oral diseases.

Overall, the studies included in this Research Topic expand our knowledge of the latest development of protein chemistry and enzymology research in a variety of biological processes, something that would not have been possible without the state-of-the-art analytic techniques and research platforms, which have been widely adopted in the last decade and will continue to contribute future studies in the field of protein chemistry and enzymology.

